# Implications of Transition in Communication Channel Profiles Before and After the COVID-19 Crisis

**DOI:** 10.1093/geroni/igaf122.2804

**Published:** 2025-12-31

**Authors:** Ha Young Choi, Jung Yeon Suh, Shannon Mejia

**Affiliations:** University of Illinois Urbana-Champaign, Urbana, Illinois, United States; University of Illinois Urbana-Champaign, Champaign, Illinois, United States; University of Illinois Urbana-Champaign, Champaign, Illinois, United States

## Abstract

This study examines transitions in older adults’ communication channel patterns during the COVID-19 crisis and investigates whether these transitions moderate the relationship between pre-pandemic social support and post-pandemic loneliness. Using Health and Retirement Study data (n = 2,830; aged 50-97), latent profile analysis identified four communication profiles in 2018: (1) in-person/phone contact with family, (2) social media (SNS) dominant, (3) email dominant, and (4) high interaction across all channels. By 2022, the in-person/phone profile was replaced by a “least interaction” profile. The other three profiles persisted. Latent transition analysis estimated the probability of stability and change in profile membership between the two time points. In-person/phone profile mostly transitioned to the least interaction profile. The high interaction profile largely remained stable. SNS profile was the least stable where members were most likely distributed to all other profiles. Email profile transitioned to all, except the least interaction, profiles. Path analyses tested, for each profile, the implications of change versus stability for the protective effects of 2018 social support for 2022 loneliness. Across all profiles, membership stability maintained the protective effects of social support on loneliness. However, friend support’s effect disappeared when transitioning from in-person/call to email profiles. Child support’s effect vanished when transitioning from SNS to least interaction profiles. Family support’s effect disappeared when moving from high to least interaction profiles. These findings suggest the pandemic significantly altered older adults’ communication patterns, with implications for mental health. Interventions should strengthen diverse social support networks and provide targeted assistance with digital communication adoption.

